# True Croup and Calomel Sublimation

**Published:** 1893-11

**Authors:** 


TRUE CROUP AND CALOMEL SUBLIMATION.—We make the following extract from an editorial in the *Northwestern Lancet:*

The use of calomel in diphtheria and croup has long been popular, but hitherto principally as a remedy by mouth, in which way it has been given in surprisingly large amounts, to give a grain every hour for a whole day being nothing unusual. The rule for its administration has been to continue it until there were produced copious and frequent greenish passages from the bowels, on the theory that the disease is largely eliminated by the liver, whose activity it is the object of the calomel treatment to increase.

Before the days of germs and ptomaines calomel had long been a favorite drug in the treatment of croup, its supposed power of checking exudation warranted the hope that through its constitutional effect it might limit the formation of the croupous membrane in the larynx. The latest method of treatment, that of sublimation, resorts to the old drug and has for its object to hold in check the formation of the membrane, but it seeks to accomplish this by bringing the calomel directly into contact with the exudation by inhalation instead of indirectly through the general system.

In speaking of the sublimation treatment as the latest method of handling croup, it was not intended to imply fhat there was anything very new about it ; in fact, it is nearly twenty years since it was first employed and described by Dr. Corbin, of Brooklyn. But it is only lately that a sufficient number of cases where this treatment was employed has been collected to give anything like a fair estimate of its value. These cases were gathered together by Dr. Wm. Madden and are described in a paper published in the August number of the *Brooklyn Medical Journal.* In answer to his inquiries he received replies from two hundred and forty-two physicians, who reported five hundred and five cases of true croup treated by calomel sublimation; of these cases, two hundred and seventy-five, or about 54.5 per cent. recovered. Of those that recovered, twenty-nine were treated by tracheotomy or intubation as well as by the calomel, leaving 48.7 per cent. who recovered after treatment by the sublimation alone. That this is a large percentage of recovery from true croup needs no further proof than an examination of the health report of St. Paul, where cases of membranous croup are reported as such, and where it will be found from the records that the mortality is very high; in the years 1891 and 1892, for instance, whose records are at hand, there are reported eighty-seven cases of membranous croup with seventy-six deaths, or more than eighty-seven per cent. In this connection the matter of diagnosis is of course of great importance. Dr. Madden gives no assurance upon the subject, but states simply that the physicians who reported the cases were nearly all from Brooklyn and New York.

The method of employing calomel sublimation is so simple that it needs no detailed description. By means of a sheet a tent is formed over the bed or crib to confine the fumes of the drug. The quantity of calomel used for each sublimation should be from half a drachm to a drachm. This is placed upon a piece of thin sheet iron, such as the cover of a tin spice box and heat is applied by means of an alcohol lamp, the sublimation being accomplished as quickly as possible. The treatment is to be repeated at varying intervals according to the amount of difficulty in breathing, sometimes half hourly and sometimes only once in two or three hours. The administration of a few grains of calomel in divided doses at the onset will also be a help. Of course this treatment does not stand in the way ol intubation or tracheotomy, and it may be necessary to continue the sublimations after the operation if there is any obstruction from formation of the membrane in the trachea.—*Southern Prac.*

